# Estimating food consumption, micronutrient intake and the contribution of large-scale food fortification to micronutrient adequacy in Tanzania

**DOI:** 10.1017/S136898002400199X

**Published:** 2024-11-11

**Authors:** Rie Goto, Liberty Mlambo, Lucia Segovia De La Revilla, Aleswa Swai, Hoyce Mshida, Alex Amos, Emilian Karugendo, Gareth Osman, Kevin Tang, Thomas Codd, Christopher Chagumaira, Elaine L Ferguson, E Louise Ander, Theresia Jumbe, Ray Masumo, Omar Dary, Jennifer Yourkavitch, Sarah Pedersen, Germana H Leyna, Monica Woldt, Edward JM Joy

**Affiliations:** 1 Faculty of Epidemiology and Population Health, London School of Hygiene & Tropical Medicine, London, UK; 2 School of Biosciences, University of Nottingham, Loughborough, UK; 3 Tanzania Food and Nutrition Centre, Dar es Salaam, Tanzania; 4 National Bureau of Statistics, Ministry of Finance & Planning, Dodoma, Tanzania; 5 Department of Human Nutrition and Health, Bunda College, Lilongwe University of Agriculture and Natural Resources, Lilongwe, Malawi; 6 World Food Programme, Rome, Italy; 7 Centre for Environmental Geochemistry, British Geological Survey, Nottinghamshire, UK; 8 USAID Advancing Nutrition, Dar es Salaam, Tanzania; 9 USAID, Bureau for Global Health, Washington, DC, USA; 10 USAID Advancing Nutrition, Arlington, VA, USA; 11 Results for Development, Washington, DC, USA; 12 Bureau for Resilience, Environment, and Food Security, USAID, Washington, DC, USA; 13 Muhimbili University of Health and Allied Sciences, Dar es Salaam, Tanzania; 14 Helen Keller International, Washington, DC, USA

**Keywords:** Household consumption and expenditure survey, Large-scale food fortification, Micronutrient adequacy, Tanzania

## Abstract

**Objective:**

To assess the potential contribution of large-scale food fortification (LSFF) towards meeting dietary micronutrient requirements in Tanzania.

**Design:**

We used household food consumption data from the National Panel Survey 2014–15 to estimate fortifiable food vehicle coverage and consumption (standardised using the adult female equivalent approach) and the prevalence at risk of inadequate apparent intake of five micronutrients included in Tanzania’s fortification legislation. We modelled four LSFF scenarios: no fortification, status quo (i.e. compliance with current fortification contents) and full fortification with and without maize flour fortification.

**Setting:**

Tanzania.

**Participants:**

A nationally representative sample of 3290 Tanzanian households.

**Results:**

The coverage of edible oils and maize and wheat flours (including products of wheat flour and oil such as bread and cakes) was high, with 91 percent, 88 percent and 53 percent of households consuming these commodities, respectively. We estimated that vitamin A-fortified oil could reduce the prevalence of inadequate apparent intake of vitamin A (retinol activity equivalent) from 92 percent without LSFF to 80 percent with LSFF at current fortification levels. Low industry LSFF compliance of flour fortification limits the contribution of other micronutrients, but a hypothetical full fortification scenario shows that LSFF of cereal flours could substantially reduce the prevalence at risk of inadequate intakes of iron, zinc, folate and vitamin B_12_.

**Conclusions:**

The current Tanzania LSFF programme likely contributes to reducing vitamin A inadequacy. Policies that support increased compliance could improve the supply of multiple nutrients, but the prominence of small-scale maize mills restricts this theoretical benefit.

Micronutrient deficiencies are a serious and widespread public health problem especially in low- and middle-income countries (LMIC)^(1–3)^. Deficiencies in a single or multiple micronutrients can contribute to complications in the perinatal period; poor child growth and cognitive development; increased risk of maternal and child illness, infections and in extreme cases death and long-term consequences such as decreased school performance and work productivity and weakened economic development^(2)^.

Inadequate dietary intakes of micronutrients is a common underlying cause of micronutrient undernutrition^(2)^. Widespread micronutrient deficiencies have been indicated previously in Tanzania in localised studies. In a cross-sectional study of school-aged children in two rural districts of Tanzania, Gowele *et al.* reported a high prevalence of Fe (29 percent), vitamin A (25 percent) and Zn (26 percent) deficiencies based on biomarker measurements; the percentage of children consuming less than the respective recommended nutrition intakes were 95 percent for Zn, 81 percent for vitamin A and 66 percent for folate based on 24-hour dietary recall data^(4)^. In a cross-sectional study of mothers at 3-months postpartum in Dar es Salaam and Morogoro Regions of Tanzania, Bellows *et al.* reported a five percent prevalence of vitamin B_12_ deficiency, 19 percent prevalence of folate deficiency and 26 percent prevalence of Fe deficiency based on biomarker measurements^(5)^.

One cost-effective and safe strategy to improve dietary micronutrient intakes, when appropriately designed and implemented is large-scale food fortification (LSFF). LSFF involves adding vitamins and minerals to commonly consumed staple foods and condiments during their processing in formal, large industries^(6,7)^. Globally, more than 140 countries have some form of mandatory fortification of staple foods or condiments. In the past two decades, LSFF programmes have reached an increasing number of people in LMIC^(8)^. Recent systematic reviews and meta-analyses have demonstrated some effectiveness of LSFF programmes on micronutrient status for vitamin A, Fe, iodine and folate for several population groups^(7,9)^. The evidence indicates that for women and children, LSFF increased serum concentrations of retinol and Hb, as well as the median urinary iodine concentration, and decreased prevalence of neural tube defects^(7)^.

Various types and sources of data may inform the design, implementation, monitoring and evaluation of LSFF programmes and policies. National food consumption data may also be used to evaluate the contribution of LSFF to dietary micronutrient intakes and the potential for further improvements through modified program design^(10,11)^. These data would help inform evidence-based and equitable LSFF policies and programmes to promote nutritious diets and fill nutrient gaps, as well as monitor their contributions over time.

Food consumption data may be generated using individual-level quantitative dietary data to make inferences for populations or individuals depending on the design, such as quantitative 24-hour recalls^(12)^. These data can then be combined with food composition data to estimate nutrient intakes and their adequacy. However, the collection of quantitative 24-hour dietary recall data is complex, time consuming and costly, particularly for applications requiring national scale data^(13,14)^. There are few surveys in LMIC that have collected 24-hour dietary recall data^(13,14)^. However, many LMICe conduct household consumption and expenditure surveys (HCES) every 3–5 years^(13)^. These surveys, which are designed primarily to generate information on household poverty and welfare, typically collect information on food consumption at the household level^(15)^. HCES data can provide proxy measures of population-level food consumption over the previous 7 or 14 d, depending on the country, which can be used to generate insights on population-level intakes of food and nutrients^(16,17)^.

In Tanzania, 26 percent (approximately 14 million people) of the population lives below the basic need poverty line, with 43 percent of households consuming only two meals per day. Furthermore, 23 percent of households reported difficulties meeting their food needs in the previous year^(18,19)^. Persistent food insecurity combined with diets low in nutrient dense foods, such as animal-source foods, legumes and vegetables, may contribute to widespread micronutrient deficiency. As a part of wider strategies to prevent inadequate micronutrient intakes, the Government of Tanzania mandated the fortification of cooking oils with vitamin A and the fortification of wheat flour and maize flour with Fe, Zn, vitamin B_12_ and folic acid in 2021^(20)^. However, the coverage of fortifiable wheat and maize flour was low (33·1 percent for wheat flour and 2·5 percent for maize flour)^(21)^. The additional Fe supplied through fortified wheat flour was estimated at only 10 percent of the recommended intake for women of reproductive age^(21)^.

The Tanzania National Panel Survey (TNPS), which is a type of HCES, is routinely conducted (Wave 1: 2008–09, Wave 2: 2010–11, Wave 3: 2012–13, Wave 4: 2014–15 and Wave 5: 2020–21) and includes a module that captures food consumption at the household level for up to sixty food items. To the best of our knowledge, this is the first study to use TNPS data to assess apparent dietary micronutrient intakes to provide evidence for the design of LSFF policies and programs in Tanzania. The specific objectives of the study were to:

1. Estimate the reach (potential availability and use) and apparent consumption of fortifiable foods such as cooking oils, maize flour and wheat flour in Tanzania.
2. Estimate the apparent intakes of micronutrients for non-pregnant and non-lactating women aged 18–29 years from household diets and the associated percent at risk of inadequate apparent intakes.
3. Estimate the current and potential contributions of LSFF towards meeting dietary micronutrient requirements under three scenarios of fortification compliance.

## Methods

Briefly, we quantified the apparent intake of five micronutrients for households participating in the Tanzania National Panel Survey 2014–2015 Wave 4 through integration of secondary datasets on food consumption and food composition. At the time of the analysis, the TNPS Wave 4 provided the most recent nationally representative data for household food consumption in Tanzania. Household-level estimates of apparent nutrient intake were calculated and standardised using the adult female equivalent approach (AFE) and compared against harmonised average requirements (H-AR) for non-pregnant and non-lactating women aged 18–29·9 years^(22)^ to define percentage at risk of inadequate apparent nutrient intakes. These H-AR define nutrient intake requirements using the most recent evidence and are proposed as suitable to ‘assess intakes of populations for many applications in global and regional contexts’^(22)^.

### Tanzania national panel survey

The TNPS Wave 4 data were accessed via the Living Standards Measurement Study of the World Bank Microdata Library^(23)^. The TNPS is a nationally representative household survey that collects information on the living standards of the Tanzanian population, including their socio-economic characteristics, consumption, agricultural production and non-farm income generating activities. The survey is designed to follow the same households over time to track changes in living conditions. In TNPS Wave 4, a nationally representative subsample of the panel cohort was selected as part of an ‘Extended Panel’ to support longitudinal comparisons with previous survey rounds^(23)^. In addition, to refresh samples with biases from the changes in boundaries, demographics or population information and reduce the risk of selection bias escalation, a new nationally representative sample was selected, called the ‘Refresh Panel’ (*n* 3360 households), which we used for this analysis. The ‘Refresh Panel’ aimed to create a sample that was broadly representative of the current population, given that participants in the panel sample get older over successive rounds. The TNPS Wave 4 sample design has four analytical strata: Dar es Salaam, other urban areas in Tanzania Mainland, rural areas in Tanzania Mainland and Zanzibar. Within each stratum, clusters were randomly selected as primary sampling units, with the probability of selection proportional to the population size of the stratum. The survey consisted of fifty-one design strata, corresponding to a rural/urban designation for each of the twenty-five regions with Dar es Salaam as a purely urban stratum. In urban areas, clusters are equivalent to census enumeration areas (EA), while in rural areas, clusters are equivalent to villages. Eight households were randomly chosen in each cluster. During the survey, one selected EA in Dar es Salaam was deleted because the houses in the EA had been demolished to pave the way for expansion of a road. The resulting sample consists of 3352 households across 419 EAs/villages. Sixty-two households did not report any food consumption in the previous 7 d, or the household reported that no member consumed foods in the household, and these households were excluded from the subsequent analysis. Therefore, a total of 3290 households (1332 and 1958 households in urban and rural areas, respectively, and 539, 529, 1757 and 465 households from Dar es Salaam, Mainland other urban areas, Mainland rural areas and Zanzibar, respectively) were analysed.

### Food consumption data

The enumerators asked a household member (typically an adult woman) to recall foods consumed over the past 7 d by all members of the household, using a predefined list of sixty distinct food items/food groups (e.g. ‘Cooking oils’), with quantities recorded in ‘kilograms’, ‘grams’, ‘litre’, ‘millilitre’ or ‘pieces’. The source of each item consumed was recorded as purchases, own production and gifts/other sources. The module did not include foods consumed outside the home.

For the quantities of food items consumed using its water weight equivalent, the weights were adjusted for liquid food items such as cooking oils^(24)^, and the non-edible portions of foods (e.g. banana skins) were subtracted^(25–27)^. The quantities reported using the unit ‘pieces’ (e.g. ‘Eggs’, ‘Chicken and other poultry’, ‘Sweets’, ‘Maize (green, cob)’ and ‘Ripe bananas’) were estimated using Food Portion Size Databases in the Tanzania Food Composition Table^(27)^ and converted to metric units (i.e. grams).

### Food composition data

National and regional food composition tables (FCT) were reviewed for use in the current study. The selection of food composition table nutrient values to match with each food item was prioritised based on the number of available foods and relevant nutrients (including moisture content) and geographic and cultural relevance. Thus, the primary source of food matches was the Kenya FCT^(25)^, followed by West Africa^(26)^, United States^(28)^ and Tanzania FCT^(27)^. Notably, the Tanzania FCT does not report moisture content, and concentration values for several nutrients were implausible, so that FCT was ranked fourth and used with caution. For the food items consumed that were reported in the predefined list as multiple food items (e.g. ‘Onions, tomatoes, carrots and green peppers, other viungo’), the individual foods were matched to their respective counterpart in the FCT, and the weighted mean of the nutrient values was calculated. The weights were based on the frequency of consumption reported in the Tanzania 2018 Household Budget Survey. Detailed information regarding the food matching protocol can be found in the open access repository (https://doi.org/10.17037/DATA.00004293).

To calculate the consumption of wheat flour, the quantity of wheat flour, both direct and as an ingredient in processed foods (i.e. wheat flour equivalents) such as ‘Breads’, ‘Buns, cakes and biscuits’ and ‘Sweets’, each were derived from a recipe and ingredients dataset compiled for Tanzania under the USAID Advancing Nutrition project (personal communication; Dr. Zo Rambeloson, USAID Advancing Nutrition consultant, September 29, 2023). Similarly, the consumption of cooking oil was calculated from ‘Sweets’ (i.e. sweet bakery products such as African donuts), ‘Buns, cakes and biscuits’ and ‘Breads’. Recipe and ingredient data are reported in see online supplementary material, Supplementary Table 1.

### Calculation of nutrient supplies and standardisation using the adult female equivalent approach

Nutrients estimated as consumed were quantified at the household level as the sum of the quantity of each nutrient from each food item quantity multiplied by its matched composition value. Household-level nutrient supplies were standardised for comparability across households using the AFE approach. Similar to the adult male equivalent approach^(29)^, the AFE approach divides household-level nutrient supplies by the sum of AFEs based on energy requirements. The resultant unit is the supply of a nutrient per day per AFE, and this can be compared against thresholds of requirements for adult women across households. We used the AFE rather than adult male equivalent metric, to facilitate comparison against nutrient density estimates (from other studies), which are typically defined for adult women rather than men due to their higher critical nutrient density thresholds, and therefore greater risk of inadequate intake in women^(30)^.

The AFE approach first involved calculating the total number of AFE for each household based on a reference value of estimated energy requirement of each member of the family, and divided by the energy requirement of a non-pregnant and non-lactating female aged 18–29·9 years. The energy requirements of household members were calculated according to their age and gender, estimating the activity level as ‘active or moderately active lifestyle’ (i.e. physical activity level (PAL) 1·85)^(31)^. The average weights used to calculate estimated energy requirements were obtained from anthropometric measurements in Wave 4 for females aged 0–50 years and males aged 0–15 years. Since anthropometric data for other age groups were not collected in TNPS Wave 4, the average weights of adult males (above 18 years of age) were estimated from the global database of estimated height and BMI^(32)^. For males aged 16 and 17 years, weights were estimated at two midpoints between the weights of 15 and 18 years of age. The weight for females above 50 years was derived from the average weight of females aged 19–49 years measured in TNPS Wave 4. Energy requirements were estimated from non-breastmilk foods for children below 24 months by subtracting the energy from breast milk, using the energy needed from complementary foods in LMIC context (i.e. infants aged 0–2 months: 0 kilocalories (kcal)/d, 3–5 months: 76 kcal/d, 6–8 months: 269 kcal/d, 9–11 months: 451 kcal/d, 12–23 months: 746 kcal/d, estimating ‘average’ energy intakes from breastmilk)^(33)^. Additional energy requirements of 500 kcal/d for lactating women were calculated as follows: women identified in the survey as being the biological mother of a child below 24 months of age were assumed as lactating women^(34)^. This assumption was necessary because there is no information on lactation or pregnancy status of participants in TNPS Wave 4.

### Estimating the adequacy of micronutrient intakes

The fixed cut-point approach was used to define the adequacy of apparent intakes at the household level for Zn, vitamin A, folate and vitamin B_12_. Requirement values were derived from the H-AR of females aged 18–29·9 years for each nutrient^(22)^. The H-AR is the intake of a nutrient that would meet the needs of half of healthy individuals in a specific life-stage group. The H-AR were as follows: Zn 10·2 mg, vitamin A 490 µg (retinol activity equivalents (RAE)), folate 250 µg (dietary folate equivalents (DFE)) and vitamin B_12_ 2 µg^(22)^. Requirements for Fe and Zn assumed ‘low absorption’ and ‘unrefined’ diets, respectively. The full probability approach was used to estimate the percentage of menstruating women at risk of inadequate Fe intakes (with 5 percent bioavailability) in women^(6)^. It provides the estimated prevalence of Fe inadequacy (data not provided on the estimated range of prevalence).

We also estimated the ‘micronutrient gap’ by subtracting the H-AR for each micronutrient from the apparent intake at the 25^th^ percentile, to provide an approximation of the additional dietary supply of micronutrients required from fortification. The 25^th^ percentile of intakes was used, instead of an alternative percentile, as recommended for programmatic uses given the approximate nature of HCES intake data^(12)^.

### *Estimating the risk of excess micronutrient intake in* large-scale food fortification

The fixed cut-point approach was used to estimate the prevalence of excess apparent intakes at the household level for Fe, Zn, vitamin A (only retinyl esters or preformed vitamin A) and folic acid. The risk of excessive intakes of folate and vitamin B_12_ was not considered due to their low potential for toxicity and for vitamin B_12_, the body does not store excess amounts^(35,36)^. The harmonised upper limit values (H-UL), for the other nutrients, were used for females aged 18–29·9 years for each nutrient^(22)^. The cut-off values of H-ULs were as follows: Fe 45 mg, Zn 25 mg (European Food Safety Authority (EFSA)) and 40 mg (Institute of Medicine (IOM) of the US National Academies), vitamin A 3000 µg retinol and 1000 µg folic acid.

### Fortification scenarios

In 2021, the Tanzania Bureau of Standards was mandated to oversee food fortification (as it does with other food-related regulations) under the Finance Act 2019, and they use the East African standards to set fortificant specifications and contents^(20)^. The standards stipulate that cooking oils should be fortified with vitamin A, with 20–40 mg RAE per kg in a retinyl palmitate form, to obtain a mean content of 25 mg/kg RE. For wheat flour and maize flour, the legislation stipulates the addition of Fe (33 mg/kg in the form of ferrous fumarate), Zn (44 mg/kg), vitamin B_12_ (0·013 mg/kg) and folic acid (2 mg/kg, which is equal to 3·4 DFE).

In the current study, four fortification scenarios were defined to generate insights on the current and potential contribution of the national LSFF programmes towards meeting dietary requirements for these five micronutrients. To assess the current contribution of LSFF, we first defined a ‘no fortification scenario’ in which we assumed potentially -fortifiable foods contained ‘natural’ concentrations of nutrients only, as reported in FCTs and considering no fortification. We then defined a ‘status quo’ scenario, which was our best attempt to quantify apparent nutrient intakes given the current mean contents due to fortification that are observed, based on compliance with fortification standards. A market-level assessment^(37)^ reported that only 28 percent of sampled cooking oils were fortified with vitamin A within the legislated range. Similarly, 47 percent of wheat flour was within the legislated range for Fe fortification, and none of the maize flour samples appeared to be fortified. The latter is not surprising, given that the majority of maize in Tanzania is milled at local facilities where fortification equipment is unlikely to be installed.

Two hypothetical ‘full fortification’ scenarios were then defined, to estimate the potential contribution of food fortification: (a) if all cooking oil, maize flour and wheat flour were fortified according to the specified standards and (b) if only cooking oils and wheat flour were fortified, excluding maize flour due to reach constraints. In the modelling, we assumed that all cooking oils, maize flour and wheat flour reported as consumed, in a household, were potentially fortifiable, thereby modelling, for fortification of these food vehicles, the upper limit of potential effectiveness.

The ‘status quo’ and ‘full fortification’ scenarios assumed using the values of (A) the proportion of mandatory fortification levels in 100 g of product, (B) the proportion of samples fortified to standard and (C) the expected proportion of vitamins lost between the point of production, sale and consumption^(6,17)^. Table 1 describes the parameter values of the food fortification scenarios modelled. The fortification parameters were calculated as follows:

- Status quo = (A × B) × (1–C)
- Full fortification = A × (1–C)

**Table 1:** The parameters of food fortification in scenarios of status quo and full fortification

		Mandatory fortification in 100 g of product (A)	Proportion of samples fortified to standard (B)	Proportion of expected losses up to home (C)	Calculated concentration of fortificant added to 100 g of product	
					Status quo	Full fortification
Cooking oils	Vitamin A, µg RAE	2500	0·28	0·30	490	1750
Maize flour	Iron, mg	3·3	0	0	0	3·3
	Zn, mg	4·4	0	0	0	4·4
	Folic acid, µg^*^	200	0	0·20	0	160
	Folate, DFE, µg^*^	340	0	0·20	0	272
	Vitamin B_12_, µg	1·3	0	0·15	0	1·1
Wheat flour	Fe, mg	3·3	0·47	0	1·55	3·3
	Zn, mg	4·4	0·47	0	2·07	4·4
	Folic acid, µg^*^	200	0	0·20	0	160
	Folate, DFE, µg^*^	340	0	0·20	0	272
	Vitamin B_12_, µg	1·3	0·47	0·15	0·52	1·1

* 2 mg/kg of folic acid is equivalent of 3.4 mg/kg folate; the value of folic acid was used to estimate the risk of excess intakes and folate DFE was used to estimate dietary contribution.

The contribution of fortificants via wheat flour and cooking oil in products such as ‘Bread’, ‘Buns, cakes and biscuits’ and ‘Sweets’ was calculated according to recipe data (see online supplementary material, Supplementary Table 1), weighted by the ingredients of component items and included in the modelling of fortification scenarios. We did not include ‘Macaroni and spaghetti’ in this calculation because we lacked information on the amount of wheat flour produced in Tanzania that was used in the macaroni and spaghetti consumed in the country.

### Data analysis

The distribution of the consumption quantities was log-transformed prior to identifying outlying values, as these were right-skewed. Outliers, which were defined as quantities >3 standard deviations from the median and missing data (0·2 percentage of the total food consumption values), were converted to the median consumption quantity of consuming households prior to further analysis.

The percentages of households reporting consumption of food fortification vehicles and the quantities consumed were reported as the median value among consumers. The reduction of the inadequacy of micronutrient intakes was calculated without changes to the proportion of the population that did not consume those foods.

Data analysis was conducted in the RStudio (RStudio 2023·06·0 Build 421, Posit Software, PBC)^(38)^ and used several R packages: *tidyverse* ^(39)^ for data manipulation and creating graphics and *survey* ^(40)^ and *srvyr* ^(41)^ for analysing complex survey samples and calculating summary statistics of the survey data. The R code for the analysis is available open access at the following repository (https://doi.org/10.17037/DATA.00004293).

## Results

### Apparent consumption of fortifiable foods at the household level

Table 2 shows the percentages of households reporting consumption of potential fortifiable food vehicles and the quantities consumed. Nationally, about 90 percent of households consumed cooking oil and maize flour. However, maize flour is processed locally in small-scale facilities that are unlikely to be reached by LSFF programmes. A little over half of households at the national level consumed wheat flour, although this was 80 % among urban households. Frequencies of consumption of cooking oils and maize flour were slightly higher in urban areas than rural areas, whereas the frequency of the consumption of wheat flour was nearly double in urban areas than rural areas. Among the consumers, the median daily consumption per AFE of cooking oils and wheat flour was 1·7 and 1·9 times higher in urban areas (30 *v*. 17 g/d and 71 *v*. 38 g/d per AFE, respectively) than rural areas, respectively, however, maize flour consumption was 1·6 times higher in rural areas than urban areas (296 *v*. 183 g/d per AFE, respectively).

**Table 2:** Percentage of households consuming potential fortifiable foods included in the national food fortification program and the median (interquartile range (IQR)) of consumption quantity among consumers by national, urban and rural areas (in grams/d per AFE)

	Cooking oils			Maize flour^*^			Wheat flour		
	%	Median	Interquartile range (IQR))	%	Median	Interquartile range (IQR))	%	Median	Interquartile range (IQR))
National	91·1	21·0	11·8–34·6	88·0	247·9	150·2–401·3	52·9	54·3	24·5–100·5
Urban	96·7	30·4	19·2–46·1	92·6	182·5	117·5–276·9	80·1	71·1	39·7–114·7
Rural	88·3	17·3	9·6–28·0	85·6	295·5	180·4–463·0	39·7	38·2	16·5–74·0

* Although maize flour is widely consumed, the majority is produced by artisanal operations and therefore difficult to be considered as LSFF.

Table 3 provides information on the percentage of households reporting consumption of food fortification vehicles and the quantities consumed in Dar es Salaam, other mainland urban areas, mainland rural areas and Zanzibar. Over 86 percent of households in all areas reported consumption of cooking oils, with the highest percentage in Dar es Salaam (99 percent), and the lowest in Zanzibar (86 percent). Reported maize flour consumption was high in the Mainland (87–95 percent) and much lower in Zanzibar (54 percent). Coverage of wheat flour consumption was 90 percent in Dar es Salaam, followed by Zanzibar (84 percent), lower in other Mainland urban areas (72 percent) and much lower in rural areas (39 percent). The consumption of cooking oil per AFE was twice as high in Dar es Salaam compared to the lowest consumption in Mainland rural areas (median of 36 and 17 g/d per AFE, respectively). Median maize flour consumption was highest in Mainland rural (302 g/d per AFE) and lowest in Zanzibar (57 g/d per AFE); for wheat flour consumption was the opposite, with median consumption highest in Zanzibar (116 g/d per AFE) and lowest in Mainland rural areas (34 g/d per AFE equivalent), about one-third of that in Zanzibar.

**Table 3:** Percentage of households consuming food vehicles included in the national food fortification program and the median (interquartile range) of consumption quantity among consumers by analytical strata (in grams/d per AFE)

	Cooking Oils			Maize Flour			Wheat Flour		
	%	Median	Interquartile range (IQR))	%	Median	Interquartile range (IQR))	%	Median	Interquartile range (IQR))
Dar es Salaam	98·6	35·6	23·6–50·2	94·9	147·2	101·1–209·7	89·6	79·8	51·9–123·2
Mainland other urban	95·6	27·5	16·8–43·6	92·8	221·7	142·1–322·6	72·3	61·6	32·5–104·4
Mainland rural	88·5	17·3	9·6–27·9	86·5	301·7	187·4–464·2	38·5	34·3	15·4–68·8
Zanzibar	85·8	19·7	9·8–33·4	54·3	56·9	32·6–103·1	83·6	116·0	64·9–172·0

See online supplementary material, Supplementary Table 2 reports the frequencies and amount of apparent food consumption per day per AFE of the sixty food items in TNPS.

### Apparent micronutrient adequacy in different population strata in Tanzania

Nationally, under the ‘no fortification scenario’, the median apparent intake of vitamin A was estimated to be less than half the H-AR for females aged 18–29·9 years (Table 4). The median apparent intake of Fe was below the H-AR. Conversely, the median apparent intake of folate was above the H-AR, except in Zanzibar. Zanzibar showed the lowest estimated nutrient intakes among the four strata for the assessed micronutrients, except vitamin B_12_ intake. Estimated vitamin A intake in Zanzibar was approximately half the national median value.

**Table 4:** The estimated daily apparent intake of iron, zinc, vitamin a, folate and vitamin B_12_ per AFE, under the no fortification scenario, by national, urban, and rural areas and four analytical strata

		Median	Interquartile range (IQR))
Fe (mg)	National	20·3	15·3–27·6
	Urban	19·5	14·7–26·1
	Rural	20·9	15·6–28·4
	Dar es Salaam	18·6	14·9–23·4
	Mainland other urban	20·3	14·8–27·6
	Mainland rural	21·1	16·0–28·6
	Zanzibar	14·1	10·1–18·8
Zn (mg)	National	11·0	8·4–15·0
	Urban	11·5	8·6–15·5
	Rural	11·1	8·3–14·9
	Dar es Salaam	10·1	8·2–12·8
	Mainland other urban	12·2	8·7–16·7
	Mainland rural	11·6	8·7–15·6
	Zanzibar	8·8	7·0–11·4
Vitamin A (RAE, µg)	National	201·3	124·5–317·0
	Urban	254·3	159·1–375·0
	Rural	179·2	112·7–279·6
	Dar es Salaam	260·5	186·5–363·4
	Mainland other urban	254·8	146·7–392·9
	Mainland rural	182·1	114·2–283·7
	Zanzibar	116·6	75·2–190·8
Folate (DFE, µg)	National	431·0	307·5–612·9
	Urban	380·2	276·7–518·9
	Rural	472·0	334·4–657·6
	Dar es Salaam	361·3	265·6–468·6
	Mainland other urban	406·8	297·7–564·9
	Mainland rural	477·2	342·4–662·6
	Zanzibar	223·5	148·7–324·9
Vitamin B_12_ (µg)	National	2·1	0·5–5·6
	Urban	2·7	1·1–6·4
	Rural	1·8	0·4–5·2
	Dar es Salaam	2·1	1·0–3·5
	Mainland other urban	3·6	1·2–8·3
	Mainland rural	1·7	0·4–5·3
	Zanzibar	2·0	1·3–3·4

### Current and potential contributions of large-scale food fortification towards meeting dietary micronutrient requirements

The prevalence of inadequate apparent micronutrient intakes was calculated nationally and stratified by urban/rural residence and analytical strata (Table 5). See online supplementary material, Supplementary Table 3 reports the estimated daily apparent intakes of Fe, Zn, vitamin A, folate and vitamin B_12_ per AFE.

**Table 5:** Prevalence (%) of micronutrient inadequacy based on four scenarios of LSFF: (a) no fortification, (b) status quo, (c) full fortification coverage and (d) full fortification coverage without maize flour fortification*, nationally and by residence and four analytical strata

		Prevalence (%) at risk of inadequate apparent intake (95 % CI)							
		No fortification		Status quo		Full fortification		Full fortification (without maize flour fortification^*^)	
		%	95 % CI	%	95 % CI	%	95 % CI	%	95 % CI
Iron^†^	National	74·7		72·6		50·8		70·4	
	Urban	79·5		76·1		57·0		72·2	
	Rural	71·5		70·3		46·6		69·1	
	Dar es Salaam	80·1		76·2		56·0		71·8	
	Mainland other urban	73·2		70·6		47·3		68·3	
	Mainland rural	69·5		68·7		43·3		67·9	
	Zanzibar	90·0		85·8		77·0		80·2	
Zn	National	40·6	38·6, 47·2	35·5	33·5, 37·5	8·8	7·4, 10·4	31·7	29·8, 33·7
	Urban	43·2	39·8, 46·5	33·2	30·1, 36·5	6·2	4·5, 8·3	26·4	23·7, 29·4
	Rural	39·3	36·8, 42·0	36·6	34·1, 39·2	10·2	8·3, 12·4	34·3	31·9, 36·8
	Dar es Salaam	52·2	47·2, 57·2	33·6	28·9, 38·7	6·5	4·6, 9·0	24·5	20·9, 28·6
	Mainland other urban	36·4	32·0, 41·1	32·7	28·5, 37·2	5·4	3·2, 8·9	27·8	23·8, 32·0
	Mainland rural	38·7	36·0, 41·4	36·4	33·8, 39·1	9·8	7·9, 12·1	34·5	32·0, 37·1
	Zanzibar	63·4	57·2, 69·2	41·3	35·9, 46·9	23·0	17·4, 29·6	28·0	22·2, 34·7
Vitamin A	National	92·4	90·9, 93·6	79·6	77·7, 81·4	45·7	43·4, 48·0	45·7	43·4, 48·0
	Urban	88·0	84·7, 90·7	66·2	62·4, 69·7	24·5	21·1, 28·1	24·5	21·1, 28·1
	Rural	94·5	93·0, 95·7	86·3	84·1, 88·2	56·2	53·3, 59·1	56·2	53·3, 59·1
	Dar es Salaam	90·1	86·6, 92·8	64·1	59·3, 68·7	16·3	13·3, 19·8	16·3	13·3, 19·8
	Mainland other urban	86·4	81·2, 90·3	66·7	61·1, 71·8	28·7	23·6, 34·4	28·7	23·6, 34·4
	Mainland rural	94·4	92·9, 95·6	86·0	83·8, 88·8	55·9	52·9, 58·9	55·9	52·9, 58·9
	Zanzibar	98·2	96·1, 99·1	92·6	88·5, 95·3	61·8	55·6, 67·6	61·8	55·6, 67·6
Folate	National	15·0	13·5, 16·5	11·9	10·6, 13·2	2·5	2·0, 3·2	10·4	8·4, 12·7
	Urban	19·2	16·6, 22·1	13·2	11·2, 15·5	3·0	2·1, 4·1	10·0	8·6, 11·8
	Rural	12·8	11·2, 14·8	11·2	9·6, 13·0	2·3	1·6, 3·1	10·7	9·2, 12·4
	Dar es Salaam	20·6	17·1, 24·5	11·9	9·6, 14·6	3·1	1·9, 4·8	8·3	6·2, 10·0
	Mainland other urban	16·7	13·2, 21·0	13·3	10·4, 16·8	2·4	1·4, 4·2	11·2	8·4, 14·9
	Mainland rural	11·6	9·9, 13·6	10·4	8·8, 12·2	1·7	1·1, 2·6	9·5	8·0, 11·3
	Zanzibar	55·1	49·5, 60·7	35·9	30·5, 41·8	18·6	14·3, 23·6	26·3	21·8, 31·4
Vitamin B_12_	National	49·0	46·7, 51·3	46·0	43·7, 48·4	11·1	9·6, 13·8	43·0	40·7, 45·4
	Urban	40·6	36·7, 44·6	34·6	30·7, 38·8	7·0	5·0, 9·7	28·7	24·9, 32·8
	Rural	53·2	50·4, 56·0	51·7	48·8, 54·6	13·1	11·2, 15·4	50·2	47·4, 53·0
	Dar es Salaam	49·1	44·3, 53·8	39·3	34·0, 44·9	6·4	4·5, 9·0	29·4	24·5, 34·8
	Mainland other urban	34·6	29·1, 40·5	31·6	26·2, 37·6	7·0	4·2, 11·5	28·4	23·0, 34·5
	Mainland rural	53·3	50·4, 56·2	52·3	49·3, 55·2	13·0	10·9, 15·3	51·0	48·0, 53·9
	Zanzibar	50·9	43·9, 57·8	33·9	27·9, 40·5	18·2	13·2, 24·5	25·0	19·4, 31·6

* As maize flour is mostly produced in artisanal and not formal mill processing facilities, it is very difficult to consider the fortification of this food as LSFF because fortification is going to be difficult and unsustainable.

† Note that we cannot estimate the 95 percent CI for the prevalence of iron inadequacy given the need to use the full probability approach.

Under the ‘no fortification scenario’, with contents of nutrients only supplied by the usual diet without food fortification, more than 90 percent of households nationally had inadequate apparent intakes of vitamin A. Three quarters of households had inadequate apparent intakes of Fe, and nearly half of households had inadequate apparent intakes of vitamin B_12_ and Zn, but only 15 percent of households had inadequate apparent intakes of folate. Compared with urban areas, rural areas showed a higher prevalence of inadequate apparent intakes of vitamin A and B_12_, but a lower percentage at risk of inadequacy for Fe, Zn and folate.

The prevalence of apparent inadequacies under the no fortification scenario were greatest in Zanzibar. For example, in Zanzibar, the estimated prevalence at risk of inadequate apparent intake of folate under the no fortification scenario was 4·5 times greater than the prevalence in Mainland rural areas and 1·6 greater for Zn. Zanzibar showed a large nutrient gap compared with other strata except vitamin B_12_ (see online supplementary material, Supplementary Table 4).

Current food fortification (‘status quo’) could reduce the prevalence at risk of inadequate apparent intakes of vitamin A, through addition of this nutrient only to oil, from 88 percent to 66 percent among urban participants, and from 95 percent to 86 percent among rural participants. Moving from the status quo to a full fortification scenario could achieve further reductions in the prevalence at risk of inadequate apparent intakes; vitamin A inadequacy could reduce to 25 percent and 56 percent among urban and rural households, respectively. Zn inadequacy could reduce from about 43 percent to 33 percent and 39 percent to 37 percent in urban and rural areas, respectively, and to 6 percent among urban households and 10 percent among rural households when full fortification is considered, and to 26 and 34 percent, respectively, when maize flour fortification is excluded. Fe inadequacy under ‘status quo’ could reduce from about 80 percent to 76 percent and 72 percent to 70 percent among urban and rural households, respectively. Folate inadequacy could reduce from about 19 percent to 13 percent and 13 percent to 11 percent among urban and rural households, respectively, under the ‘status quo’ scenario, and to 3 and 2 percent, respectively, under the full fortification scenario, and to 10 and 11 percent, respectively, excluding maize flour fortification. Vitamin B_12_ inadequacy could reduce from about 41 percent to 35 percent and 53 percent to 52 percent among urban and rural households, respectively, under a ‘status-quo’ condition, and to 7 and 13 percent, respectively, under a full fortification scenario, and to 29 and 50 percent, without maize flour fortification. In summary, reduction in the inadequacy of the micronutrients added to the flours is important but primarily when maize flour fortification is considered, but this is a very optimistic scenario as this staple is difficult to be fortified in a sustainable manner. This highlights the need to differentiate the populations that are consuming maize flour produced by large and formal factories from those that are not.

When compared to the status quo scenario, the percentage point reduction in the prevalence at risk of inadequate apparent micronutrient intakes under the full fortification scenario generally less in Zanzibar than in other strata, except for vitamin A and folate. With full fortification, we estimate that the prevalence of inadequate intake would still be highest in Zanzibar compared with other areas of Tanzania for the five micronutrients (for example, the nutrient gap of Fe remained negative with full fortification in Zanzibar – see online supplementary material, Supplementary Table 4). In Zanzibar, the prevalence of inadequate intakes would be about twice as high for Fe and Zn and over 10 times as high for folate, when compared with the rural mainland.

### Current and estimated risk of excess micronutrient intakes from large-scale food fortification

The estimation of excess apparent micronutrient intakes was calculated nationally and stratified by urban/rural residence and in four analytical strata (Table 6).

**Table 6:** Estimated proportion (%) of excess intakes of iron, zinc, vitamin A and folic acid based on four large-scale food fortification scenarios: (a) no fortification, (b) status quo, (c) full fortification coverage and (d) full fortification coverage without maize flour fortification^*^, nationally and by residence and four analytical strata

		Estimated proportion (%) of excess intake (95 % CI)							
		No fortification		Status quo		Full fortification		Full fortification (without maize flour fortification^*^)	
		%	95 % CI	%	95 % CI	%	95 % CI	%	95 % CI
Fe	National	6·4	5·3, 7·9	6·9	5·7, 8·3	18·7	16·9, 20·7	7·4	6·1, 8·8
	Urban	4·6	2·8, 7·3	5·4	3·6, 8·1	14·3	11·2, 18·2	6·2	4·3, 8·9
	Rural	7·4	5·9, 9·2	7·6	6·1, 9·4	20·9	18·8, 23·2	8·0	6·5, 9·8
	Dar es Salaam	2·6	1·6, 4·4	3·3	1·9, 5·6	6·6	4·6, 9·4	3·7	2·3, 6·1
	Mainland other urban	5·9	3·2, 10·6	6·9	4·1, 11·4	19·7	14·6, 25·9	7·9	5·0, 12·3
	Mainland rural	7·6	6·1, 9·4	7·8	6·3, 9·7	21·4	19·2, 23·8	8·1	6·6, 10·0
	Zanzibar	0·4	0·1, 2·3	0·7	0·3, 2·2	2·9	1·3, 6·2	1·6	0·7, 3·6
Zn (EFSA)	National	4·3	3·6, 5·2	6·1	5·3, 7·1	45·2	42·9, 47·5	7·6	6·6, 8·7
	Urban	3·5	2·5, 4·9	6·4	5·0, 8·0	37·6	34·9, 40·5	8·5	6·9, 10·5
	Rural	4·7	3·7, 5·9	6·0	5·0, 7·2	49·0	45·9, 52·1	7·1	5·9, 8·5
	Dar es Salaam	1·8	1·0, 3·3	3·3	1·9, 5·5	27·3	23·4, 31·5	6·3	4·5, 8·8
	Mainland other urban	4·6	3·1, 6·9	8·4	6·4, 11·0	45·2	41·4, 49·1	10·0	7·6, 12·9
	Mainland rural	4·7	3·6, 5·9	6·1	5·0, 7·3	50·0	46·8, 53·2	7·0	5·8, 8·4
	Zanzibar	2·0	0·7, 5·6	3·8	2·0, 7·0	14·7	10·4, 20·2	9·4	6·4, 13·6
Zn (IOM)	National	0·5	0·3, 1·0	0·7	0·4, 1·2	12·3	11·0, 13·7	1·0	0·7, 1·5
	Urban	0·5	0·2, 1·5	0·7	0·3, 1·8	8·0	6·3, 10·2	0·9	0·5, 1·5
	Rural	0·5	0·2, 1·1	0·7	0·4, 1·3	14·4	12·8, 16·3	1·3	0·7, 2·3
	Dar es Salaam	0·1	0·02, 0·9	0·3	0·08, 1·24	3·2	1·9, 5·5	0·7	0·3, 1·8
	Mainland other urban	0·7	0·2, 2·5	1·0	0·3, 2·8	11·3	8·6, 14·7	1·6	0·7, 3·4
	Mainland rural	0·5	0·3, 1·2	0·7	0·4, 1·4	14·8	13·1, 16·7	0·9	0·5, 1·5
	Zanzibar	0		0·5	0·1, 2·2	3·3	1·6, 6·7	1·2	0·5, 2·9
Vitamin A (as retinyl esters or preformed vitamin A)	National	0		0		0		0	
	Urban	0		0		0		0	
	Rural	0		0		0		0	
	Dar es Salaam	0		0		0		0	
	Mainland other urban	0		0		0		0	
	Mainland rural	0		0		0		0	
	Zanzibar	0		0		0		0	
Folic acid	National	0		0		0		0	
	Urban	0		0		0		0	
	Rural	0		0		0		0	
	Dar es Salaam	0		0		0		0	
	Mainland other urban	0		0		0		0	
	Mainland rural	0		0		0		0	
	Zanzibar	0		0		0		0	

* As maize flour is mostly produced in artisanal and not formal mill processing facilities, it is very difficult to consider the fortification of this food as LSFF because fortification is going to be difficult and unsustainable.

The estimated risk of excess intakes for Fe and Zn was low in the models of ‘no fortification’, ‘status quo’ and ‘full fortification’ when maize flour fortification was excluded. However, in the presence of maize flour fortification following the actual standard, excess of Fe intake was determined as 19 percent (strata range from 3 to 21 percent), and Zn (using the IOM UL criterion) showed 12 percent (strata range from 3 to 15 percent) excess intake. If Zn intake is judged using the EFSA criteria, the proportion of excessive intakes would be larger. No excess intakes of vitamin A (i.e. retinyl esters or preformed vitamin A) and folic acid from the fortified foods were estimated in any of the models. Among regions, the highest excess intakes for Fe and Zn were found in mainland rural areas, principally due to the high consumption of maize flour.

## Discussion

The current study quantified apparent micronutrient intakes using national HCES data to estimate the prevalence of dietary micronutrient inadequacy and explore the potential contribution of LSFF to mitigate micronutrient inadequacy in Tanzania. In the absence of food fortification, the prevalence at risk of inadequate apparent intakes was very high (>60 percent nationally) for vitamin A and Fe, high (30–60 percent nationally) for Zn and vitamin B_12_ and low to moderate (10–30 percent) for folate.

A large proportion of households (particularly in urban areas) reported consumption of food items covered by current LSFF legislation (i.e. cooking oils, wheat flour and maize flour). However, the percentage of food samples collected at households that met legislated fortification levels were low (less than half of wheat flour samples, less than one-third of cooking oil samples and no maize flour samples)^(37)^; hence, low compliance is likely to hinder the effectiveness of LSFF in Tanzania. Moreover, a large proportion of maize flour is produced by informal processing facilities and therefore very difficult to be fortified in an efficient and sustainable manner. The reduction in prevalence of inadequate apparent intakes between the no fortification and status quo scenarios was small for the micronutrients added to cereal flours (e.g. ≤5 percentage point reduction for Fe, Zn, folate and vitamin B_12_ at national levels). However, the current fortification of oil with vitamin A program is likely to play an important role in reducing vitamin A inadequacy, with a 13-percentage point reduction in the prevalence at risk of inadequate apparent intakes at the national level.

Increasing compliance could deliver substantial further improvements in the supply of multiple nutrients. For example, at the national level, moving from the status quo to the full fortification scenario (including maize flour fortification) could reduce the prevalence at risk of inadequate apparent intakes by one-third (from 73 percent to 51 percent) for Fe, and two-thirds (from 46 percent to 11 percent) for vitamin B_12_. Generally, the potential effectiveness of LSFF (defined by change in prevalence of inadequate apparent intakes) under the status quo scenario, or the full compliance but excluding maize flour, appears to be greater in Zanzibar and urban areas compared to the Mainland rural areas, due to greater consumption of cooking oil and wheat flour in terms of coverage and amount consumed per day per AFE. Nevertheless, the potential effectiveness of moving from the status quo scenario to full fortification, when including maize flour, was lower in Zanzibar than other strata for Fe, Zn and vitamin B_12_. This was primarily due to the low reported consumption of maize flour in Zanzibar. While the ‘full fortification’ scenario may be considered an ideal scenario, greater than 95 percent of maize flour in Tanzania comes from micro-, small- and medium-scale milling facilities^(42)^, so the theoretical benefit observed under this scenario is restricted, and it may be challenging to increase access to fortification technologies for these small-scale processing facilities due to costs and difficulties in monitoring safety and compliance. Complementary strategies including dietary diversification and biofortification will remain important, especially for the mainland rural areas.

The daily apparent intakes per AFE of vitamin A under a ‘no fortification’ scenario were 22 percent lower than values reported in Malawi, in a study using HCES data collected during 2016–2017^(17)^, whereas apparent intakes of Fe, Zn, folate and vitamin B_12_ were approximately 5 percent, 25 percent, 35 percent and 57 percent greater in Tanzania than in Malawi. This difference was due to the higher reported consumption of animal-source foods and vegetables in Tanzania than in Malawi. For example, the median consumption of animal-source foods in the current study was 62 g/d per AFE (data not shown), whereas in Malawi the quantity was 15 g/d per AFE^(43)^. Additionally, HCES data in Malawi indicated an underestimation of food intakes, with an estimated energy intake per day per AFE of 2112 kcal (IQR 1367 kcal per day per AFE)^(43)^. In comparison, estimated energy intakes in Tanzania were 2492 kcal (IQR 1331, data not shown). A potential underestimation of food consumption in Malawi would translate to lower nutrient intakes, including for Fe and Zn. The Malawi study was similar to the current study, in observing a significant effect of current fortification (status quo scenario) on apparent intakes of vitamin A, but not other micronutrients, due to low compliance with legislation. In Malawi, like in Tanzania, the staple cereal maize is dominant and almost all consumption is derived from micro- and small-scale mills.

We also estimated the risk of excess intakes in various fortification models. Low prevalence of excess intakes was found in the status quo and full fortification without maize flour fortification scenarios. However, the full fortification model, which included maize flour fortification, showed a high risk of excess intakes for Fe and Zn. The prevalence of excess intake was generally higher in rural areas compared with urban areas. On a national level, the apparent estimated maize consumption was about 250 g/d per AFE, with rural areas consuming about 1·6 times more maize flour than urban areas (183 *v*. 296 g/d per AFE, respectively).

The estimated prevalence of excess Zn intakes should be interpreted with caution because the current value of the UL is widely understood to be lower than necessary^(44)^ (e.g. usual dietary Zn intakes often exceed nearly double the UL among US children^(45,46)^). Furthermore, the large difference between the two UL for Zn (EFSA: 25 mg/d and IOM: 40 mg/d for females aged 18–29·9 years) suggests the need for further discussion. Use of the IOM value reduced the prevalence of excess Zn intake from 45 percent to 12 percent compared with the EFSA value. To indicate the safety of micronutrient intakes, we calculated the difference between the H-UL and the apparent intake of Zn at the 75^th^ percentile^(12)^. The gap was –7·4 mg/d using the EFSA UL and +7·6 mg/d using the IOM UL at national level under the full fortification scenario, with a positive difference indicating safe intakes (data not shown).

By using the TNPS dataset as a source of information on food consumption, this study provides a national picture of apparent intake of micronutrients and associated risks of inadequate intake using a low cost and fast secondary analysis of data. The design of the survey also enabled us to reveal important sub-national insights, including differences in risks of inadequate micronutrient intakes between four analytical strata and between urban and rural populations. A further strength is the inclusion and modelling of fortification scenarios, which provide novel effectiveness estimates of the current LSFF programmes and reveal the large potential benefit to population nutrition that could be achieved by increasing compliance with current LSFF legislation.

There are several limitations to the current study, and findings should be interpreted with due caution. In the absence of a national survey of individual-level dietary data, this study used household-level data from 7-day recall. Limitations of this approach have been covered extensively in previous studies^(17,47,48)^. We analysed the TNPS Wave 4 conducted in 2014–2015 and patterns of food consumption may have changed over the past decade, including due to economic and cultural drivers. However, this was the most recent national data on food consumption available at the time of the study, and the current study methods can be applied to future survey data when these become available. The HCES provides only a proxy of food consumption at the household level, which is neither directly reported nor observed. TNPS captures consumption of sixty food items; more detailed resolution of food items would likely improve the accuracy of apparent nutrient intake estimates, although a longer food item list may contribute to respondent fatigue. Disaggregation of cooking oils would be particularly valuable for exploring the current and potential contributions of LSFF.

The current study assumed that foods, and therefore nutrients, were distributed within the household according to household members’ energy requirements. This assumption was necessary, given that consumption was recalled at the household level, but it fails to capture potential inequities in distribution, for example, due to gender or age^(49)^. Furthermore, the model was not adjusted for the additional nutrient requirements arising from pregnancy as pregnancy status of household members was not recorded in the TNPS. Micronutrient composition of the fortified food items was derived from relevant food composition datasets with a set of assumptions around fortification compliance. We employed a simple modelling framework to assume that all consumption of flours and oils was potentially fortifiable^(17)^. Our full fortification scenarios explored the hypothetical upper limits to the improvements in nutrient adequacy that could be achieved if all cooking oils, maize flour and wheat flour were fortified. An alternative approach that warrants further investigation is to assume fortification only of the quantity of food items recorded by households as ‘purchased’ but not from ‘own production’ or ‘gifts’. Although nearly all cooking oils (100 percent) and wheat flour (98 percent) were purchased, 38 percent of households consumed non-purchased maize flour and so the full fortification scenario with maize flour included would reduce in potential effectiveness. A limitation of this approach, however, is that ‘purchased’ maize flour does not necessarily mean that it was milled at a centralised and formal facility; similarly ‘gifted’ maize flour may have been.

As is typical of dietary intake studies, single values of food micronutrient composition were assumed to apply to the whole population, which was necessary due to limitations in available data, but this will fail to capture geographic variation in ‘natural’ composition of foods^(50)^ and in fortification compliance. Notably, due to data quality and availability, we largely had to rely on food composition data collated for countries and regions outside of Tanzania. Finally, the characterisation and parameterisation of our fortification scenarios weree necessarily simplistic, for example, we did not explore a scenario that maintained the percentage of food items that were fortifiable while varying the compliance with standards.

Nonetheless, findings of the current study provide a valuable perspective on risks of inadequate micronutrient intakes in Tanzania and could be used to inform LSFF programme and policy decisions when used in conjunction with data from other studies, including individual-level dietary nutrient intakes and biomarker status. Notably, the Tanzania DHS is due to report biomarker data for several micronutrients including Fe, vitamin A, folate and vitamin B_12_ from approximately 6000 women and children within the next 1–2 years. This survey will also analyse the Fe content of wheat and maize flours and the retinyl palmitate content of edible oils. There are opportunities to improve the value of future TNPS rounds for generating population nutrition insights, including through expanding the list of food items (i.e. de-concatenate items such as ‘Mangoes, avocados and other fruits’ and types of cooking oils and include a category for orange-fleshed sweet potato), and collecting staple food samples from participant households for direct measurement of micronutrient composition. Additionally, results from individual-level dietary survey data collected from all household members are needed to inform assumptions on intra-household food distribution.

## Supporting information

Goto et al. supplementary material Goto et al. supplementary material

## Supplementary material

For supplementary material accompanying this paper visit https://doi.org/10.1017/S136898002400199X
