# Supplementary material for: Estimating food consumption, micronutrient intake and the contribution of large-scale food fortification to micronutrient adequacy in Tanzania
Source: Public Health Nutr. 2024 Nov 11;27(1):e230. doi: 10.1017/S136898002400199X (PMC11645117; doi:10.1017/S136898002400199X)
Supplement: Goto et al. supplementary material [file S136898002400199Xsup001.docx]

Supplementary Table 1. The percentage of wheat flour and oil in selected food items

|  |  | Wheat flour (%) | Mean (%) | Oils (%) | Mean (%) |
| --- | --- | --- | --- | --- | --- |
| Buns, cakes, and biscuits | Chapati with oil | 89.67 | 84.5 | 9.35 | 7.1 |
|  | Cake | 88.82 |  | 4.87 |  |
|  | Biscuit | 74.90 |  | - |  |
| Bread | Bread roll | 93.04 | 86.0 | 4.87 | 4.9 |
|  | Bread, brown | 88.82 |  | 4.87 |  |
|  | Bread, white | 76.17 |  | 4.85 |  |
| Sweets | African donut | 84.21 | 82.0 | 13.92 | 12.2 |
|  | Wheat fritter, sweet | 79.87 |  | 10.54 |  |

NB: The proportion of ingredients were obtained from personal communication, Dr. Zo Rambeloson, USAID Advancing Nutrition consultant, September 29, 2023.

Supplementary Table 2. Percentage of households consuming 60 food items and the median (interquartile range) of consumption quantity among consumers (grams/day per AFE), national

| Food items | Frequency (%) | Apparent consumption quantity (grams/day per AFE) (median, IQR) |
| --- | --- | --- |
| Salt | 99.2 | 7.3 (5.6–11.0) |
| Onions, tomatoes, carrots and green pepper, other viungo | 91.6 | 58.4 (31.6–101.5) |
| Cooking oil | 89.4 | 18.0 (10.6–29.2) |
| Maize (flour) | 88.0 | 247.5 (150.2–401.3) |
| Spinach, cabbage, and other green vegetables | 82.0 | 39.7 (21.6–72.2) |
| Sugar | 75.9 | 31.7 (21.4–45.4) |
| Peas, beans, lentils, and other pulses | 74.6 | 49.7 (28.6–81.3) |
| Rice (husked) | 62.2 | 131.5 (75.0–208.4) |
| Tea dry | 62.0 | 1.1 (0.7–1.6) |
| Fresh fish and seafood (including dagaa) | 51.7 | 14.2 (6.4–29.6) |
| Beef including minced sausage | 40.5 | 34.4 (23.0–59.4) |
| Buns, cakes, and biscuits | 35.0 | 33.8 (16.8–66.3) |
| Mangoes, avocadoes, and other fruits | 34.5 | 46.1 (23.6–97.7) |
| Dried/salted/canned fish and seafood (incl. dagaa) | 33.3 | 13.2 (6.9–25.6) |
| Cooking bananas, plantains | 30.7 | 65.1 (38.2–133.9) |
| Ripe bananas | 29.6 | 29.5 (15.6–52.6) |
| Irish potatoes | 28.9 | 52.7 (31.3–97.5) |
| Groundnuts in shell/shelled | 28.5 | 27.0 (13.3–49.6) |
| Fresh milk | 27.9 | 110.9 (54.6–219.8) |
| Sweet potatoes | 27.4 | 94.8 (55.3–183.3) |
| Citrus fruits (oranges, lemon, tangerines, etc.) | 25.2 | 22.3 (9.3–50.7) |
| Cassava fresh | 24.5 | 88.7 (53.5–147.8) |
| Coconuts (mature/immature) | 22.0 | 37.3 (19.1–65.7) |
| Bread | 21.6 | 42.6 (25.0–75.4) |
| Eggs | 19.9 | 8.4 (5.1–14.0) |
| Cassava dry/flour | 18.5 | 133.7 (60.3–267.4) |
| Maize (grain) | 18.1 | 62.5 (39.4–131.1) |
| Bottled/canned soft drinks (soda, juice, water) | 17.4 | 56.4 (34.0–92.7) |
| Chicken and other poultry | 17.2 | 35.7 (24.0–58.5) |
| Wheat flour | 17.1 | 49.2 (32.3–77.9) |
| Canned, dried, and wild vegetables | 13.8 | 25.8 (12.8–54.8) |
| Sugarcane | 13.6 | 5.8 (2.9–11.6) |
| Milk products (like cream, cheese, yoghurt, etc.) | 13.2 | 63.9 (30.1–126.1) |
| Maize (green, cob) | 12.9 | 59.2 (28.8–111.0) |
| Goat meat | 12.7 | 30.5 (17.9–57.3) |
| Millet and sorghum (flour) | 11.1 | 65.5 (32.3–169.6) |
| Other spices | 9.0 | 5.0 (2.1–10.8) |
| Macaroni, spaghetti | 7.0 | 29.4 (20.7–50.5) |
| Honey, syrups, jams, marmalade, jellies, canned fruits | 6.1 | 17.8 (8.2–40.1) |
| Sweets | 5.6 | 1.1 (0.5–2.2) |
| Yams/cocoyams | 5.5 | 86.4 (48.7–141.4) |
| Pork including sausages and bacon | 4.0 | 32.8 (20.9–47.1) |
| Local brews | 3.8 | 88.1 (46.3–218.2) |
| Butter, margarine, ghee, and other fat products | 3.2 | 11.9 (6.1–18.1) |
| Cashew, almonds, and other nuts | 2.2 | 27.2 (12.8–55.9) |
| Other cereal products | 1.8 | 43.2 (29.6–78.7) |
| Coffee and cocoa | 1.7 | 1.8 (1.0–5.5) |
| Rice (paddy) | 1.5 | 272.9 (112.1–436.2) |
| Other starches | 1.2 | 105.2 (59.8–191.9) |
| Millet and sorghum (grain) | 1.1 | 56.6 (30.3–146.7) |
| Bottled beer | 1.1 | 65.2 (39.3–87.4) |
| Canned milk/milk powder | 0.9 | 16.0 (8.9–22.8) |
| Other domestic/wild meat products | 0.7 | 37.7 (14.8–58.7) |
| Wheat, barley grain, and other cereals | 0.6 | 22.5 (8.2–65.5) |
| Wild birds and insects | 0.4 | 18.6 (7.4–60.0) |
| Other raw materials for drinks | 0.4 | 0.9 (0.5–6.4) |
| Seeds and products from nuts/seeds (excl. cooking oil) | 0.2 | 1.4 (0.1–2.6) |
| Wine and spirits | 0.2 | 9.8 (5.3–60.3) |
| Prepared tea, coffee^*^ | 0.2 | 26.9 (20.8–33.5) |
| Package fish | 0.03 | 24.4, 34.1^*^ |

*IQR was not calculated for package fish (n=2)

Supplementary Table 3. The estimated daily apparent intakes of iron, zinc, vitamin A, folate, and vitamin B12 per AFE based on four large-scale food fortification scenarios: a) no fortification, b) status quo, c) full fortification coverage, and d) full fortification without maize flour fortification, nationally and by residence and analytical strata

|  |  | No fortification | Status quo | Full fortification | Full fortification (without maize flour fortification) |
| --- | --- | --- | --- | --- | --- |
| Iron, mg | National | 20.3 (15.3–27.6) | 21.0 (15.6–28.4) | 29.8 (22.0–40.7) | 21.6 (16.0–29.3) |
|  | Urban | 19.5 (14.7–26.1) | 20.5 (15.3–27.4) | 27.9 (21.4–37.1) | 21.6 (16.1–28.9) |
|  | Rural | 20.9 (15.6–28.4) | 21.3 (15.7–29.0) | 31.0 (22.4–42.2) | 21.6 (16.0–29.4) |
|  | Dar es Salaam | 18.6 (14.9–23.4) | 19.6 (15.7–24.8) | 26.3 (21.0–32.5) | 21.1 (16.6–26.5) |
|  | Mainland other urban | 20.3 (14.8–27.6) | 21.5 (15.3–28.7) | 29.9 (22.1–40.2) | 22.3 (15.9–30.5) |
|  | Mainland rural | 21.1 (16.0–28.6) | 21.5 (15.9–29.2) | 31.4 (23.1–42.4) | 21.8 (16.1–29.6) |
|  | Zanzibar | 14.1 (10.1–18.8) | 15.5 (11.3–20.9) | 18.6 (13.0–25.6) | 17.4 (12.3–23.6) |
| Zinc, mg | National | 11.3 (8.5–15.4) | 12.2 (9.0–16.4) | 23.5 (16.7–32.4) | 12.8 (9.4–17.5) |
|  | Urban | 11.1 (8.6–15.0) | 12.4 (9.2–16.8) | 22.1 (17.0–29.3) | 14.0 (10.0–18.9) |
|  | Rural | 11.5 (8.4–15.5) | 12.0 (8.9–16.2) | 24.8 (16.6–33.9) | 12.4 (9.1–16.8) |
|  | Dar es Salaam | 10.1 (8.2–12.8) | 11.8 (9.1–14.9) | 20.2 (16.3–25.7) | 13.5 (10.3–17.4) |
|  | Mainland other urban | 12.2 (8.7–16.7) | 13.5 (9.3–18.3) | 23.9 (18.4–32.0) | 14.6 (9.8–20.3) |
|  | Mainland rural | 11.6 (8.7–15.6) | 12.0 (8.9–16.2) | 25.0 (16.9–34.1) | 12.4 (9.1–16.8) |
|  | Zanzibar | 8.8 (7.0–11.4) | 10.9 (8.3–14.1) | 14.6 (10.5–20.4) | 12.9 (9.5–18.4) |
| Vitamin A, µg RAE | National | 201.3 (124.5–317.0) | 297.5 (188.4–452.7) | 532.0 (321.6–813.8) | 532.0 (321.6–813.8) |
|  | Urban | 254.3 (159.1–375.0) | 397.9 (273.5–567.4) | 741.7 (495.6–1060.8) | 741.7 (495.6–1060.8) |
|  | Rural | 179.2 (112.7–279.6) | 258.6 (165.5–387.4) | 444.9 (273.4–670.6) | 444.9 (273.4–670.6) |
|  | Dar es Salaam | 260.5 (186.5–363.4) | 418.0 (314.8–565.9) | 815.0 (590.3–1069.0) | 815.0 (590.3–1069.0) |
|  | Mainland other urban | 254.8 (146.7–392.9) | 389.3 (253.0–577.2) | 694.1 (454.7–1060.8) | 694.1 (454.7–1060.8) |
|  | Mainland rural | 182.1 (114.2–283.7) | 261.1 (167.0–388.1) | 447.1 (277.9–671.2) | 447.1 (277.9–671.2) |
|  | Zanzibar | 116.6 (75.2–190.8) | 195.6 (107.9–308.2) | 376.6 (172.8–644.1) | 376.6 (172.8–644.1) |
| Folate, µg DFE | National | 431.0 (307.5–612.9) | 472.1 (338.6–654.3) | 1158.1 (798.1–1619.1) | 506.3 (360.6–707.7) |
|  | Urban | 380.2 (276.7–518.9) | 442.9 (324.7–608.5) | 1016.7 (747.6–1374.4) | 507.8 (359.4–714.9) |
|  | Rural | 472.0 (334.4–657.6) | 484.9 (348.6–675.1) | 1246.7 (832.8–1757.1) | 506.1 (360.8–704.0) |
|  | Dar es Salaam | 361.3 (265.6–468.6) | 440.8 (336.6–575.8) | 930.7 (716.9–1237.3) | 517.0 (391.5–692.3) |
|  | Mainland other urban | 406.8 (297.7–564.9) | 450.5 (322.4–642.5) | 1067.5 (807.7–1504.8) | 497.0 (349.7–735.6) |
|  | Mainland rural | 477.2 (342.4–662.6) | 492.1 (354.3–682.3) | 1260.5 (862.3–1777.8) | 512.1 (366.5–707.1) |
|  | Zanzibar | 223.5 (148.7–324.9) | 314.4 (209.6–440.1) | 496.8 (307.9–713.5) | 390.6 (246.8–590.5) |
| Vitamin B12, µg | National | 2.1 (0.5–5.6) | 2.3 (0.7–5.9) | 5.8 (3.6–9.7) | 2.6 (0.7–6.2) |
|  | Urban | 2.7 (1.1–6.4) | 3.1 (1.4–6.8) | 5.6 (3.8–10.2) | 3.5 (1.7–7.3) |
|  | Rural | 1.8 (0.4–5.2) | 1.9 (0.4–5.4) | 5.8 (3.4–9.5) | 2.0 (0.5–5.6) |
|  | Dar es Salaam | 2.1 (1.0–3.5) | 2.5 (1.4–4.1) | 4.7 (3.5–6.7) | 3.0 (1.8–4.8) |
|  | Mainland other urban | 3.6 (1.2–8.3) | 3.9 (1.4–8.5) | 7.3 (4.1–12.2) | 4.4 (1.6–8.9) |
|  | Mainland rural | 1.7 (0.4–5.3) | 1.8 (0.4–5.5) | 5.9 (3.5–9.6) | 1.9 (0.5–5.6) |
|  | Zanzibar | 2.0 (1.3–3.4) | 2.5 (1.7–4.0) | 3.6 (2.4–5.7) | 3.3 (2.0–5.0) |

Supplementary Table 4. The nutrient gap (the difference between the apparent nutrient intake at the 25th percentile and H-AR) of iron, zinc, vitamin A, folate, and vitamin B12 based on four large-scale food fortification scenarios: a) no fortification, b) status quo, c) full fortification coverage, and d) full fortification without maize flour fortification, nationally and by residence and analytical strata

|  |  | No fortification | Status quo | Full fortification | Full fortification (without maize flour fortification) |
| --- | --- | --- | --- | --- | --- |
| Iron, mg | National | -7.1 | -6.8 | -0.4 | -6.4 |
|  | Urban | -7.7 | -7.1 | -1.0 | -6.3 |
|  | Rural | -6.8 | -6.7 | 0 | -6.4 |
|  | Dar es Salaam | -7.5 | -6.7 | -1.4 | -5.8 |
|  | Mainland other urban | -7.6 | -7.1 | -0.3 | -6.5 |
|  | Mainland rural | -6.4 | -6.4 | +0.7 | -6.3 |
|  | Zanzibar | -12.3 | -11.1 | -9.4 | -10.1 |
| Zinc, mg | National | -1.7 | -1.2 | +6.5 | -0.8 |
|  | Urban | -1.8 | -1.0 | +6.8 | -0.2 |
|  | Rural | -1.6 | -1.3 | +6.4 | -1.1 |
|  | Dar es Salaam | -2.0 | -1.1 | +6.1 | +0.1 |
|  | Mainland other urban | -1.5 | -0.9 | +8.2 | -0.4 |
|  | Mainland rural | -1.5 | -1.3 | +6.7 | -1.1 |
|  | Zanzibar | -3.2 | -1.9 | +0.3 | -0.7 |
| Vitamin A, µg RAE | National | -365.5 | -301.6 | -168.4 | -168.4 |
|  | Urban | -330.9 | -216.5 | +5.6 | +5.6 |
|  | Rural | -377.3 | -324.5 | -216.6 | -216.6 |
|  | Dar es Salaam | -303.5 | -175.2 | +100.3 | +100.3 |
|  | Mainland other urban | -343.3 | -237.0 | -35.3 | -35.3 |
|  | Mainland rural | -375.8 | -323.0 | -212.1 | -212.1 |
|  | Zanzibar | -414.8 | -382.1 | -317.2 | -317.2 |
| Folate, µg DFE | National | +57.5 | +88.6 | +548.1 | +110.6 |
|  | Urban | +26.7 | +74.7 | +497.6 | +109.4 |
|  | Rural | +84.4 | +98.6 | +582.8 | +110.8 |
|  | Dar es Salaam | +15.6 | +86.6 | +466.9 | +141.5 |
|  | Mainland other urban | +47.7 | +72.4 | +557.7 | +99.7 |
|  | Mainland rural | +92.4 | +104.3 | +612.3 | +116.5 |
|  | Zanzibar | -101.3 | -40.4 | +57.9 | -3.2 |
| Vitamin B12, µg | National | -1.5 | -1.3 | +1.6 | -1.3 |
|  | Urban | -0.9 | -0.6 | +1.8 | -0.3 |
|  | Rural | -1.6 | -1.6 | +1.4 | -1.5 |
|  | Dar es Salaam | -1.0 | -0.6 | +1.5 | -0.2 |
|  | Mainland other urban | -0.8 | -0.6 | +2.1 | -0.4 |
|  | Mainland rural | -1.6 | -1.6 | +1.5 | -1.5 |
|  | Zanzibar | -0.7 | -0.3 | +0.4 | 0 |
